# To Our Readers

**Published:** 1877-01-20

**Authors:** 


### TO OUR READERS.

It is believed that the adoption of the cash system in the future will bring a two-fold benefit: 1st, It will secure a prompt and punctual publication by the printers; and 2d, By relieving the editors from the harrassing labor of collecting, etc., will enable them to devote more attention to the preparation and selection of matter for the journal.

We do earnestly request our subscribers to save us from any future necessity of referring to the subject of dues, that those in arrears will respond without delay, and all who have sent up renewal orders will promptly remit, on the reception of this number. We hope not one will fail, as our contract with the publishers demands a strict adherence to the cash system.

